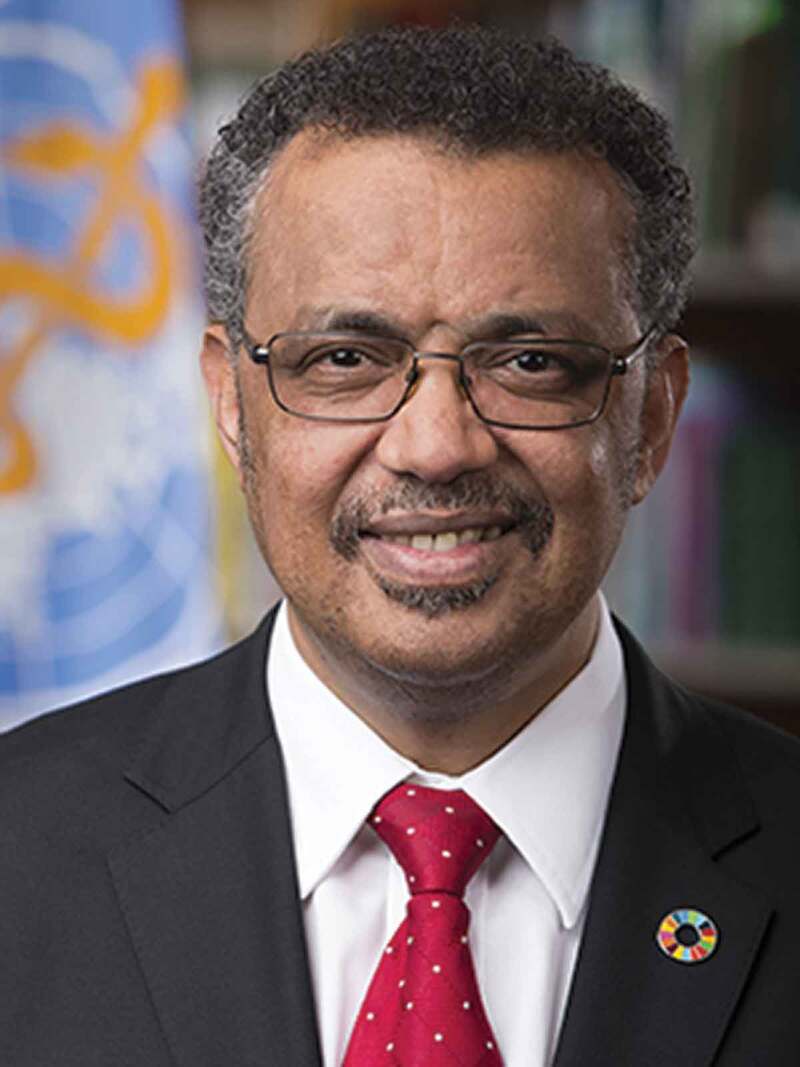# Making AMR history: a call to action

**DOI:** 10.1080/16549716.2019.1638144

**Published:** 2019-07-11

**Authors:** Tedros Adhanom Ghebreyesus

**Affiliations:** Director-General, World Health Organization, Geneva, Switzerland

Antimicrobial resistance (AMR) represents a major global health threat. Without urgent action to reverse its course, AMR could reverse a century of medical progress, cause irreparable damage to the environment, push more people into extreme poverty, imperil global health security and jeopardize progress towards the Sustainable Development Goals and the attainment of universal health coverage.

More alarmingly, new challenges are on the horizon. Recent evidence shows that AMR is emerging in conflict zones, where chronic and recurrent infections, coupled with scarce resources, can drive the emergence and spread of AMR, leading to a reservoir of resistance that will be difficult to eradicate. Studies also indicate similar trends in the emergence and spread of AMR linked to natural and man-made disasters, as well as large movements of populations and animals where organized, cross-boundary coordinated activities may be very difficult to plan.

It is very clear that AMR cannot be controlled by the health sector alone. Drug resistance recognizes no borders, as it is transmitted to humans through the food chain and through environmental degradation. All government sectors, from health to trade to agriculture, must be engaged urgently.

But managing and coordinating action across the health sector and beyond to include animal health, the environment and food production is complex and time-consuming. Unfortunately, many countries lack the human and financial resources to do this. Addressing the root causes of AMR requires an integrated ‘One Health’ approach.

Economically, the impact of AMR is immense. A recent OECD report [1] estimates that complications due to AMR could cost an average USD $3.5 billion a year across the 33 OECD countries alone. The World Bank also estimates that the impact on economic growth of antimicrobial resistance, if left unchecked, will be greater than that of the 2008 financial crisis, putting at risk up to $100 trillion of economic output by 2050 [2]. Low-and middle-income countries already bear significantly higher rates of resistance (40%-60%) compared with an average of 17% for OECD countries. If no action is taken, resistance to second- and third-line antibiotics will increase the most, and we will lose the most advanced and effective line of defense to prevent infections.

Preventing the extraordinary human, social, economic and environmental costs of AMR requires significant investments in research, as well as sustained technical, societal and political action at the highest levels of global leadership.

I therefore welcome this special issue devoted to the prevention of AMR. Sharing best practices and evidence-informed approaches to AMR control is critical to turn back the tide of this global public health crisis.

**Figure:**